# SP2509, a specific antagonist of LSD1, exhibits antiviral properties against Porcine epidemic diarrhea virus

**DOI:** 10.1186/s12917-024-04052-5

**Published:** 2024-05-10

**Authors:** Xinyu Zhao, Yuhang Zhang, Shiyin Qu, Wuyang Tang, Tianqiong He, Pishun Li, Xiaofeng Zheng

**Affiliations:** 1 College of Veterinary Medicine, Hunan Agricultural University, Changsha, 410128 China; 2 Department of Laboratory Animal Science, Central South University, Changsha, 410013 China

**Keywords:** Antiviral, Porcine epidemic diarrhea virus, SP2509

## Abstract

**Background:**

Porcine epidemic diarrhea virus (PEDV), a type of coronavirus, is one of the main pathogens that can infect pigs of all ages. It causes diarrhea and acute death of newborn piglets, resulting in massive economic losses to the worldwide swine industry. While vaccination remains the primary approach in combating PEDV, it often fails to address all the challenges posed by the infection, particularly in light of the emergence of evolving mutant strains. Therefore, there is a critical need to identify potent antiviral drugs that can effectively safeguard pigs against PEDV infection.

**Results:**

In this study, the antiviral efficacy of SP2509, a specific antagonist of Lysine-specific demethylase 1(LSD1), was evaluated in vitro. The RT-qPCR, Western blot, TCID_50,_ and IFA showed that at a concentration of 1µmol/L, SP2509 significantly inhibited PEDV infection. Additionally, viral life cycle assays showed that SP2509 operates by impeding PEDV internalization and replication rather than attachment and release. Regarding mechanism, in Huh-7 cells, knockdowns LSD1 can suppress PEDV replication. This indicated that the inhibition effect of SP2509 on PEDV largely depends on the activity of its target protein, LSD1.

**Conclusion:**

Our results in vitro show that SP2509 can inhibit PEDV infection during the internalization and replication stage and revealed a role of LSD1 as a restriction factor for PEDV. These imply that LSD1 might be a target for interfering with the viral infection, and SP2509 could be developed as an effective anti-PEDV agent.

**Supplementary Information:**

The online version contains supplementary material available at 10.1186/s12917-024-04052-5.

## Background

The Porcine epidemic diarrhea virus (PEDV), classified as an alphacoronavirus within the coronaviridae family, is a single-stranded positive-sense RNA virus encapsulated within a capsid [1]. It causes porcine epidemic diarrhea (PED), a highly contagious and acute intestinal disease that has been extensively researched [2]. Pigs of all ages can be infected by PEDV, but suckling piglets within 7 days of age show the most severe symptoms, which usually manifest as acute diarrhea, vomiting, and dehydration, and its morbidity and mortality can reach 100% [3]. PED was first recognized in England in 1971 and spread to other European countries [4]. In 2010, PED broke out in China and led to the loss of over 1 million piglets [5]. PED caused a pandemic in the United States and resulted in the death of approximately 7 million piglets and significant economic losses for the pig industry in 2013 [6, 7]. To date, the mutating PEDV has become endemic in almost all pig-rearing regions of the world, and its severity and prevalence have caused widespread concern worldwide. Currently, there is no specific therapeutic drug for PEDV, the prevention and control of PEDV is mainly based on vaccination. However, the high variability of PEDV poses a significant challenge for developing efficient vaccines for the complicated PED epidemic [8, 9]. Therefore, it is urgent to develop safe and effective drugs that can complement the vaccination approach.

Lysine-specific demethylase 1(LSD1), also known as KDM1A, is a monoamine oxidase within the cell nucleus. Its primary function involves removing methyl groups from the lysine residues at the 4th and 9th positions of histone H3 [10, 11]. Dynamic regulation of histone methylation by LSD1 has significantly influenced the activation and inhibition of gene transcription and plays a crucial role in various diseases such as cancer and viral infections [10, 12–14]. Studies have shown that SP2509, a specific LSD1 antagonist, can retard tumor cell growth by inhibiting cell proliferation and inducing cell cycle arrest [15, 16]. Furthermore, it can also induce apoptosis in tumor cells to achieve anti-tumor effects [17–19]. For antiviral functions, SP2509 can hinder herpes simplex virus type 1 (HSV-1) immediate early (IE) gene expression, DNA replication and virus production by inhibiting the LSD1-dependent demethylation of H3K9 on the promoter of HSV-1 IE gene [14, 17]. However, whether SP2509 has antiviral properties against PEDV has not been investigated. Examining SP2509's impact on PEDV proliferation and its underlying mechanism could yield valuable insights, potentially furnishing theoretical support for PEDV prevention and control strategies.

## Results

### The replication of PEDV in Vero cells

**Fig. 1:**
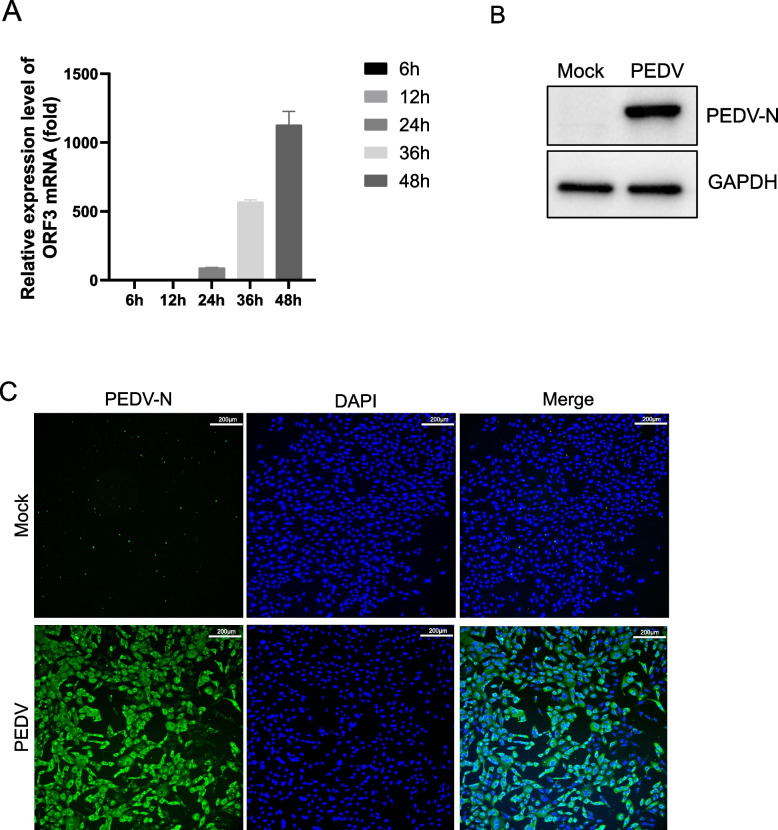
The replication of PEDV in Vero cells. **A** RT-qPCR detection of PEDV ORF3 gene mRNA levels at different times of PEDV infection. **B** Western blot to detect the expression level of N protein at 24 h of PEDV infection. **C** IFA to detect the efficiency of infection after 24 h of PEDV infection

Vero cells are widely used to propagate PEDV due to its susceptibility. Here, we evaluated the kinetics of PEDV replication in Vero cells. Vero cells were infected with PEDV at a multiplicity of infection (MOI) of 0.01 . mRNA levels of the PEDV ORF3 gene were measured using RT-qPCR and normalized against the host GAPDH gene at different time points post-infection (Fig. 1A). The PEDV N protein expression level and infection efficiency in Vero cells were assessed at 24 hpi using Western blot and IFA, respectively (Fig. 1B and C). Based on the results, PEDV replication appears to occur extensively within the infected cells.

### Cytotoxicity of the SP2509 on Vero cells

**Fig. 2:**
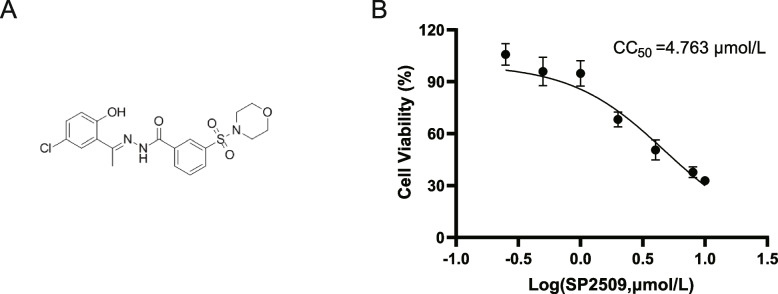
Chemical formula of SP2509 and cytotoxicity of SP2509 **A** Chemical formula of SP2509. **B** CC_50_ curves of the SP2509

The structure formula of SP2509 is shown in Fig. 2A. We first performed CCK8 assay to determine the effect of different concentrations of SP2509(ranging from 0 to 10 µmol/L: 0, 0.25, 0.5, 1.0, 2.0, 4.0, 8.0, and 10.0 µmol/L) on cell viability. The half-maximal cytopathic concentration (CC_50_) of SP2509 can be calculated as 4.763µmol/L.

We found that SP2509 was not significantly toxic to Vero cells up to 1 µmol/L (Fig. 2B). For all subsequent experiments, we therefore used SP2509 at 1 µmol/L, based on these results.

### Antiviral activity of SP2509 on Vero cells

**Fig. 3:**
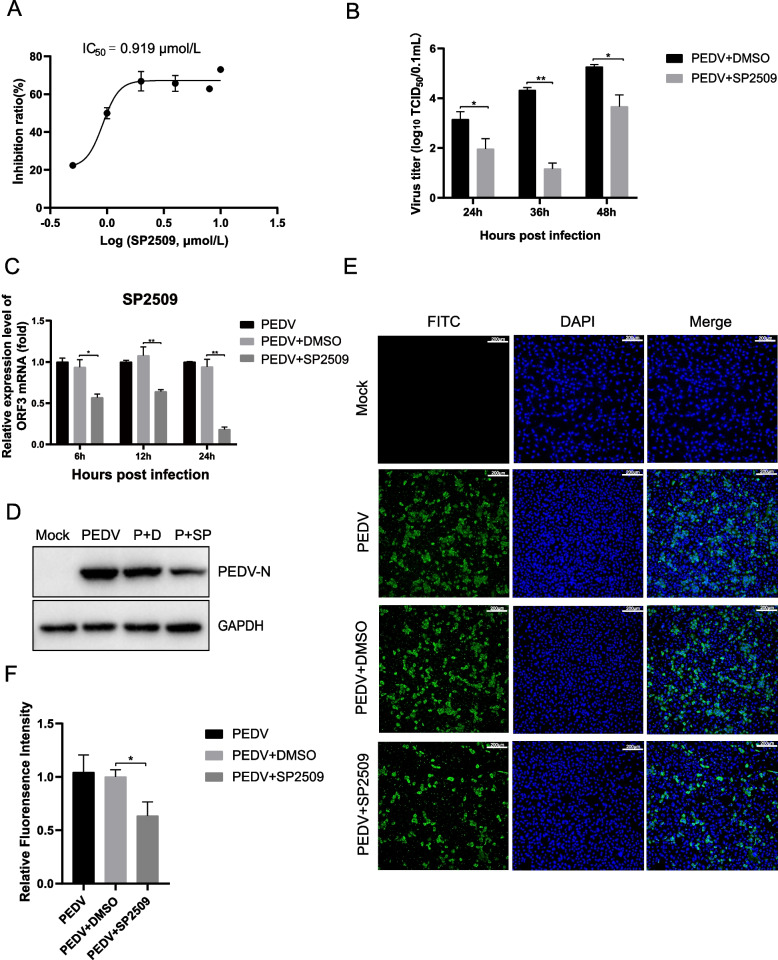
SP2509 inhibits PEDV infection in Vero cells. Vero cells were pretreated with SP2509 for 12 h, followed by mock infection or infection with PEDV (MOI = 0.01). 2 h later, cells were further cultured in viral maintenance solution in the absence or presence of SP2509. The cells were assayed for relevant indices at the indicated times after infection. **A** IC_50_ curves of the SP2509. **B** TCID_50_ analyzed the virus titer. **C** RT-qPCR analyzed the Relative RNA expression levels of PEDV ORF3. **D** The expression of PEDV N was analyzed by Western blot. **E** The inhibitory effect of SP2509 on PEDV N protein was determined by immunofluorescence assay. **F** IFA fluorescence quantification results. The experiment was performed three times independently. Differences were considered significant at * *P* < 0.05, ** *P* < 0.01, *** *P* < 0.001, **** *P* < 0.0001

The antiviral effects of SP2509 were verified by determining half maximal inhibitory concentration (IC_50_) on Vero cells. IF method was used to detect the inhibition rate of SP2509 on PEDV at different concentrations (0.5, 1.0, 2.0, 4.0, 8.0, 10.0 µmol/L), and the IC_50_ value was determined to be 0.919 µmol/L (Fig. 3A). Then, to further investigate the impact of SP2509 on PEDV infection, we infected Vero cells with PEDV(0.01MOI) and exposed the cells to 1 µmol/L SP2509 for the indicated time.

Virus supernatant was collected, and the virus titer was determined using the TCID_50_ method to assess the impact of SP2509. The results indicated that SP2509 significantly reduced the virus titer compared to the DMSO group (Fig. 3B). Then, we conducted RT-qPCR to analyze the levels of PEDV ORF3 at different time points. Our results showed that the PEDV-ORF3 expression decreased significantly after SP2509 treatment, as shown in Fig. 3C. Next, we collected cells and assessed PEDV N protein expression between the DMSO or SP2509 treated groups. Western blot showed that SP2509 decreased PEDV N protein expression compared to the DMSO group (Fig. 3D). Moreover, immunofluorescence staining indicated that the number of infected cells in the SP2509-treated group was reduced compared to the DMSO-treated group (Fig. 3E, F). In summary, the results demonstrate that a concentration of 1 µmol/L of SP2509 can effectively inhibit PEDV infection in vitro.

To test whether the antiviral effect of SP2509 is related to its target LSD1,we constructed a Huh-7 cell line capable of stably knocking down LSD1 and explored the effect of LSD1 silencing on PEDV infection. The results demonstrated that silencing LSD1 expression significantly suppressed PEDV proliferation (Supplement Figure 1) compared with the control group, which is consistent with the results of drug inhibition.

### SP2509 impaired PEDV internalization and replication instead of attachment or release

**Fig. 4:**
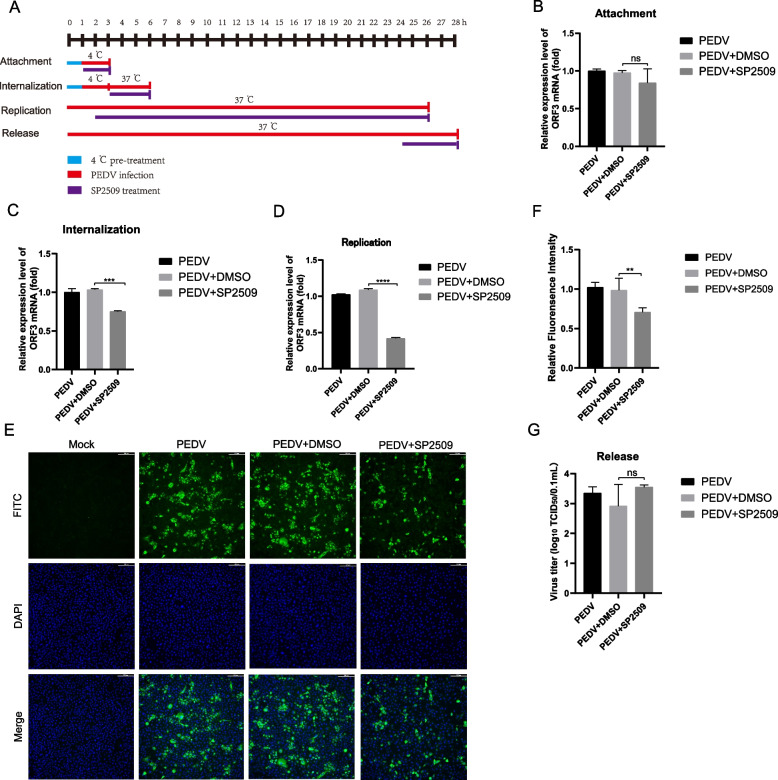
The antiviral effect of SP2509 on different infection steps of PEDV.The supernatant was replaced with SP2509 during the virus's attachment, invasion, replication, and release phases. **A** SP2509 treatment schemes. The blue bar represents 4℃ pretreatment, the red bars represent PEDV infection, the purple bars represent SP2509 treatment, and the vertical bars represent the cell collection. **B** Virus attachment assay. RT-qPCR detected PEDV-ORF3 mRNA level. **C** Virus internalization assay. PEDV-ORF3 mRNA level was detected by RT-qPCR. **D** Virus replication assay. PEDV-ORF3 mRNA level was detected by RT-qPCR. **E** IFA detection of SP2509 on PEDV replication phase. **F** IFA fluorescence quantification results. **G** Virus release assay. A virus in the supernatant was detected by TCID_50_. Data are expressed as three independent experiments. ns *P* > 0.05, * *P* < 0.05, ** *P* < 0.01, *** *P* < 0.001, **** *P* < 0.0001

To further explore the mechanism by which SP2509 inhibits PEDV infection, we conducted investigations to identify the specific stage at which SP2509 interferes with PEDV infection (Fig. 4A).

For the viral attachment assays, a mixture of 1 µmol/L SP2509 or DMSO and virus (MOI = 0.01) was mixed and added to cells, followed by incubation at 4℃ for 2 h. After extensive washing, the treated cells were collected to extract RNA to assess the effect of SP2509 on PEDV attachment through RT-qPCR. No significant difference in the mRNA level of PEDV-ORF3 was observed, indicating that SP2509 did not affect the attachment of PEDV to the Vero cell (Fig. 4B).

To assess the effect of SP2509 on PEDV internalization, Vero cells were first infected with PEDV for 2 h at 4 °C. After removing the virus inoculum, the supernatant was replaced with 1µmol/L SP2509 or DMSO, and cells were cultured at 37 °C for 3 h. RT-qPCR was then utilized to determine the influence of SP2509 on PEDV-ORF3 expression. According to the results from Fig. 4C, SP2509 impaired PEDV internalization.

Next, 1µmol/L SP2509 or DMSO was added to the supernatant during the PEDV replication stage. Based on RT-qPCR , PEDV-ORF3 was significantly lower in groups treated with SP2509 (Fig. 4D). This suggests that SP2509 can inhibit the replication phase of PEDV, which was confirmed by immunostaining results in Fig 4 E and F.

Finally, Vero cells were continuously infected with PEDV for 24 h. The viral solution was replaced with a maintenance solution, with or without SP2509. After culture at 37℃ for 4 h, the supernatant was collected, and TCID_50_ detected the virus titer. The results showed that the titer of the virus in the supernatant of the SP2509-treated group was not significantly different from that of the DMSO group. This means that SP2509 did not affect the release phase of PEDV (Fig. 4G).

Together, the above results implied that SP2509 primarily inhibited PEDV infection by inhibiting virus internalization and replication rather than attachment or release.

## Discussion

The emergence of mutant strains has exacerbated the economic losses caused by the circulation of PEDV in the global pig industry. While developing effective vaccines remains pivotal in preventing and controlling PEDV, it is likely that vaccines may not sufficiently address all challenges presented by the PEDV pandemic. Therefore, antiviral drugs are urgently needed as well to supplement the vaccination approach. To date, no drugs or compounds in clinical practice can effectively suppress PEDV, although some have been reported to have potential effects [20–22].

SP2509 is widely used as a selective LSD1 antagonist in anti-tumor studies [17], but research on the antiviral function of SP2509 has been rarely reported. In recent years, only one study showed that SP2509 can interfere with HSV-1 immediate early gene expression and DNA replication by inhibiting LSD1 activity [14]. Interestingly though, a survey of the anti-tumor activity of SP2509 reported that SP2509 could inhibit JAK/STAT3 signaling and related downstream gene expression dependent on its original target LSD1 in cancer cells [16], which in turn was tied to the innate immune response of the host cell. This led us to postulate whether this ability of SP2509 could serve as a potential mechanism for its antiviral function.

In this study, we undertook an initial exploration into the potential anti-PEDV activity of SP2509. We first created a model for PEDV infection using Vero cells and examined the cellular toxicity of SP2509. Then, we investigated the anti-PEDV activity of SP2509 and its impact on the virus life cycle. We found that SP2509 exhibited a significant antiviral effect at 1 µmol/L, mainly inhibiting the invasion and replication phases of the virus. Our work suggested that SP2509 may serve as a potential antiviral therapeutic drug.

The life cycle of a virus includes attachment, entry, transcription, replication, gene expression, assembly, maturation, and release [22]. Among them, the invasion and replication phases are crucial for the infection process. Much research has reported that inhibitors that target viruses' internalization or replication stages represent a new avenue for developing effective antiviral drugs. For instance, recent investigations have revealed that certain compounds, such as quercetin and tomatidine, can inhibit PEDV replication by targeting 3CL protease [23, 24]. Meanwhile, melatonin has also shown potential for inhibiting TGEV, PEDV, and PDCoV infections in PK15, Vero, or LLC-PK1 cells by reducing viral entry and replication [22].Furthermore, niclosamide can significantly inhibit the entry stage of PEDV by influencing viral internalization into cells [25].

In this study, SP2509 primarily acts as an antiviral effect mainly by inhibiting the invasion and replication phases of PEDV.Generally, PEDV binds to the host cell receptor, and its genome is either directly released into the cells through membrane fusion or the endosomal pathway [25–27]. The results in this paper confirm that SP2509 can inhibit the invasion phases of PEDV, suggesting that SP2509 may interact with proteins on the cell surface or viral surface to resist viral invasion. Moreover, we discovered that SP2509 impedes the replication stage of PEDV infection, indicating its possible influence on viral gene transcription. Since SP2509 is a specific inhibitor of LSD1, we speculate that the inhibition of viral replication by SP2509 may be related to the activity of LSD1. Although RNA viruses do not rely on chromatin structure and histone capsid, it is still possible that LSD1 influences RNA virus replication through demethylation of host or viral proteins [28]. For example, studies have reported that LSD1 restricts influenza A virus replication by demethylating and activating IFITM3, a host restriction factor for many RNA viruses [28]. However, the role of LSD1 in regulating PEDV replication is still unknown. To determine whether LSD1 affects PEDV replication, a Huh-7 cell line that stable knockdown LSD1 was generated. The cells were infected with PEDV, and the result showed that PEDV N protein expression significantly decreased in LSD1- knockdown cells compared with the control group. These suggested that LSD1 inhibition plays a vital role in restricting PEDV infection.

The role of LSD1 and its demethylase activity in PEDV infection remains to be further studied. One recent study showed that loss of LSD1 inhibits the nuclear factor kB-dependent inflammatory response of viral infection, enhances the IFN-independent antiviral response, and blocks the export of the virus through the restoration of lysosomal acidification. It indicates that inhibition of the LSD1 modulates the balance between inflammatory and antiviral responses against coronaviruses [29]. Therefore, we speculate that inhibition of the LSD1 may also block PEDV replication by influencing the expression of some essential genes and the intracellular immune response. However, we cannot rule out the possibility of alternative mechanisms at play that are yet to be fully elucidated. Further studies are required to explain the specific pathways through which SP2509 exerts its antiviral effects.

## Conclusion

In conclusion, our results demonstrate that SP2509 can inhibit PEDV infection during the virus’s internalization and replication stage in vitro. Moreover, the inhibition effect of SP2509 on PEDV mainly depended on inhibiting its target protein LSD1 in Huh-7 cells. This provides a theoretical basis for the clinical use of SP2509 in anti-PEDV therapy.

## Methods

### Cell culture and viral infection

African green monkey kidney cells (Vero cells), HEK293T cells and Huh-7 cells were preserved in the Hunan Engineering Technology Research Center of Veterinary Drugs (Hunan Agricultural University, Changsha, China). These cells were cultured in high-glucose Dulbecco's modified Eagle's medium (DMEM, Servicebio, China) with the addition of 10% (v/v) fetal bovine serum (FBS, Biological Industries, Israel),100 U/mL penicillin, and 100 μg/mL streptomycin (Gibco, USA). The cells were cultured in an incubator at 37 ℃ and 5% CO_2_.

The PEDV CV777 strain (Gene Bank number: AF353511) was kindly donated by Prof. Fei Liu from the School of Zoology, Nanjing Agricultural University. In all experiments, the cells were washed twice with PBS (Servicebio, China) before being infected with PEDV at a multiplicity of infection (MOI) of 0.01. After 2 h of PEDV adsorption, the unbound virus was removed by washing the cells 3 times with PBS. The cells were then cultured in DMEM containing 2% FBS at 37 °C for different times before being harvested. The viral titers were calculated using Kaerber's method and expressed as 50% tissue culture infectious doses (TCID_50_) per milliliter.

### Cell viability assay

The cytotoxic effects of SP2509 (GlpBio, USA) on Vero cells were assessed using the Cell Counting Kit-8 (CCK-8, APExBIO, USA). Briefly, 1 × 10^4^ Vero cells were seeded per well in 96-well plates containing different concentrations of SP2509. After 48 h, 10 μL of CCK-8 was added to each well, and the samples were incubated for 1~4 h. The absorbance of each well was measured at 450 nm using a microplate reader (BioTek, USA). All assays were performed in triplicate.

### Western blotting

Cells were washed twice with PBS and incubated with 2× loading buffer (0.1M Tris-HCl,4% (w/v) SDS,0.2M DTT,3mM Bromophenol blue,20% Glycerol) to lyse the cells and extract the total cellular protein. The extracted total protein was heated at 95°C for 10 min and sonicated for 10 min, after which the protein sample was separated by sodium dodecyl sulphate–polyacrylamide gel electrophoresis (SDS-PAGE) and transferred onto nitrocellulose membranes (Pall Corporation, USA). The membranes were blocked with PBS containing 5% nonfat dry milk and 0.05% Tween 20 (Sinopharm Chemical Reagent Co.Ltd., China) for 2 h at room temperature. Next, they were incubated with the primary antibody (1403113, PEDV-N Monoclonal Antibody, medgene, USA; ET1601-4, Anti-GAPDH, HUABIO, China; ER1802-12 Anti-LSD1, HUABIO, China) at room temperature for 2 h. After washing the membranes with PBS containing 0.05% Tween 20 (0.05% PBS-T), they were incubated with the corresponding secondary antibody (GB23303, HRP-labeled goat anti-rabbit IgG; GB23301, HRP-labeled goat anti-mouse, Servicebio, China) at room temperature for 1 h. Finally, the membranes were detected using a chemiluminescent imaging system (BIO-RAD, USA).

### Immunofluorescence assay (IFA)

Vero cells grown on cell slides were treated with 1 µmol/L SP2509 and subsequently infected with PEDV at a multiplicity of infection (MOI) of 0.01 for 24 h. To assess the effect of SP2509 on PEDV infection, Vero cells were pretreated with SP2509 for 12 h. After 24 h of PEDV infection in the presence of SP2509, the Vero cells were fixed with 4% paraformaldehyde (Biosharp, China) for 9 min and permeabilized with 0.5% Triton X-100 (Solarbio, China) at room temperature for 10 min and blocked with 3% bovine serum albumin (BSA, BioFroxx, China) for 1 h. The cells were then incubated with anti-PEDV-N antibody for 2 h at 37 ℃, washed with PBS three times, and incubated with a fluorescent secondary antibody (B40961, Thermo Fisher Scientific, USA) for 1 h and DAPI (Solarbio, China) for 5 min. After washing three times with PBS, fluorescence was visualized using an inverted fluorescence microscope (Leica, Germany), and the immunostaining results were quantified using Image J software (National Institutes of Health).

### Quantitative real-time PCR (RT-qPCR)

Total RNA was extracted from Vero cells using TransZol Up (TransGen Biotech, China) and reverse-transcribed into cDNA using the ToloScript RT EasyMix for qPCR (with 2-step gDNAErase-Out) (TOLOBIO, China) following the manufacturer's guidelines. 2×Q3 SYBR qPCR Master mix (Universal) (TOLOBIO, China) was used for qPCR analysis on the Real-time fluorescence quantitative PCR instrument (Roche, Switzerland). Table S1 lists the primers used for qPCR. The target gene's relative expression levels were calculated using the 2^−△△Ct^ method.

### Virus titration

Vero cell suspension was added to the 96-well plate in advance. Subsequently, the viral supernatant was gradient diluted with DMEM containing 2% FBS and added to 96-well plates. Next, the cells were incubated at 37 ℃ for 5~7 d, and the number of cell lesions was observed and recorded daily. The 50% tissue culture infectious dose (TCID_50_) was calculated by the method of Reed and Muench.

### Viral attachment, internalization, replication, and release assay

Viral attachment assay: Vero cells were cooled in advance at 4°C for 1h, and subsequently, the cells were incubated with PEDV (MOI = 0.01) in the absence or presence of 1 µmol/L SP2509 (or DMSO) at 4 °C for 2 h. After that, the cells were washed 3 times with pre-cooled PBS, and the cell lysates were harvested for RT-qPCR analysis.

Viral internalization assay: The Vero cells were pre-cooled at 4°C for 1h before incubating with PEDV (MOI = 0.01) for 2 h at the same temperature. Following this, unbound viruses were washed away with pre-cooled PBS three times. The cells were then incubated in DMEM containing 2% FBS and 1 µmol/L SP2509 (or DMSO) at 37 °C for 3 h. Finally, cell lysates were harvested for RT-qPCR analysis.

Viral replication assay: Vero cells were infected with PEDV (MOI = 0.01) and incubated at 37 °C for 2 hours. Following this, non-internalized viruses were removed by washing the cells with PBS. Then, the cells were incubated with DMEM containing 2% FBS and 1 µmol/L SP2509 (or DMSO) for 24 h at 37 °C. Cell lysates were harvested for RT-qPCR, and fixed cells were used for IFA analysis.

Viral release assay: Vero cells were infected with PEDV (MOI=0.01) for 24h at 37 °C. The cells were washed 3 times with PBS and incubated with DMEM containing 2% FBS and 1 µmol/L SP2509 (or DMSO) at 37 °C for 4 h. The supernatant was harvested for TCID_50_.

### Plasmids construction, lentivirus production and transduction

The shRNA targeting the LSD1 gene and a random sequence negative control named shNT were designed. Complementary single-stranded oligos were annealed and cloned into the pLKO.1-TRC lentivirus vector to generate shLSD1 and shNT lentivectors. All sequences used are listed in Table S2. All plasmids were verified by sequencing.

To produce lentivirus, shRNA constructs were co-transfected into 293T cells with the packaging plasmid pMD2.G and psPAX2 (Addgene, United States) using lipofiter reagents (Hanbio, China). The medium was refreshed with 2 mL advanced DMEM with 10% FBS at 6-8 h post-transfection. The culture supernatant was collected as lentivirus stock, followed by incubation for 48 h. Huh-7 cells were transduced with lentiviruses with hexadimethrine bromide (Sigma-Aldrich, United States) to enhance the infection rate. The medium was refreshed at 24 h post-infection, and stable cell lines for LSD1 knockdown were selected by using puromycin (Thermo Fisher Scientific, United States) at a concentration of 2 µg/mL. The random sequence vector (shNT) was treated as a control.

### Statistical analysis

All results are representative of 3 independent experiments. Data were presented as means ± standard deviations (SD) and analyzed with the two-tailed Student's t-test using the GraphPad Prism 8.0.2 software (GraphPad Software, USA). A *P* value of < 0.05 was considered to be a statistically significant difference. ns, *P* >0.05; *, *P* <0.05; **, *P* <0.01; ***, *P* <0.001, ****, *P* <0.0001.

### Supplementary Information

Supplementary Material 1.

Supplementary Material 2.

Supplementary Material 3.

Supplementary Material 4.

## Data Availability

All data generated or analyzed during this study are included in this published article.

## Abbreviations

PED: Porcine epidemic diarrhea
PEDV: Porcine epidemic diarrhea virus
CCK-8: Cell Counting Kit-8
RT-qPCR: Quantitative Real-Time PCR
WB: Western Blotting
IFA: Immunofluorescence Assay
TCID_50_: 50% tissue culture infective dose
HSV-1: Herpes simplex virus type 1
DMEM: Dulbecco's modified Eagle's medium
FBS: Fetal bovine serum
MOI: Multiplicity of infection
DMSO: Dimethyl sulfoxide
SD: Standard deviations
hpi: Hours post-infection
IC_50_: Half maximal inhibitory concentration
CC_50_: Half maximal cytopathic concentration
