# Supplementary figures and images for: SP2509, a specific antagonist of LSD1, exhibits antiviral properties against Porcine epidemic diarrhea virus
Source: BMC Vet Res. 2024 May 10;20:187. doi: 10.1186/s12917-024-04052-5 (PMC11084069; doi:10.1186/s12917-024-04052-5)

Fig 1B

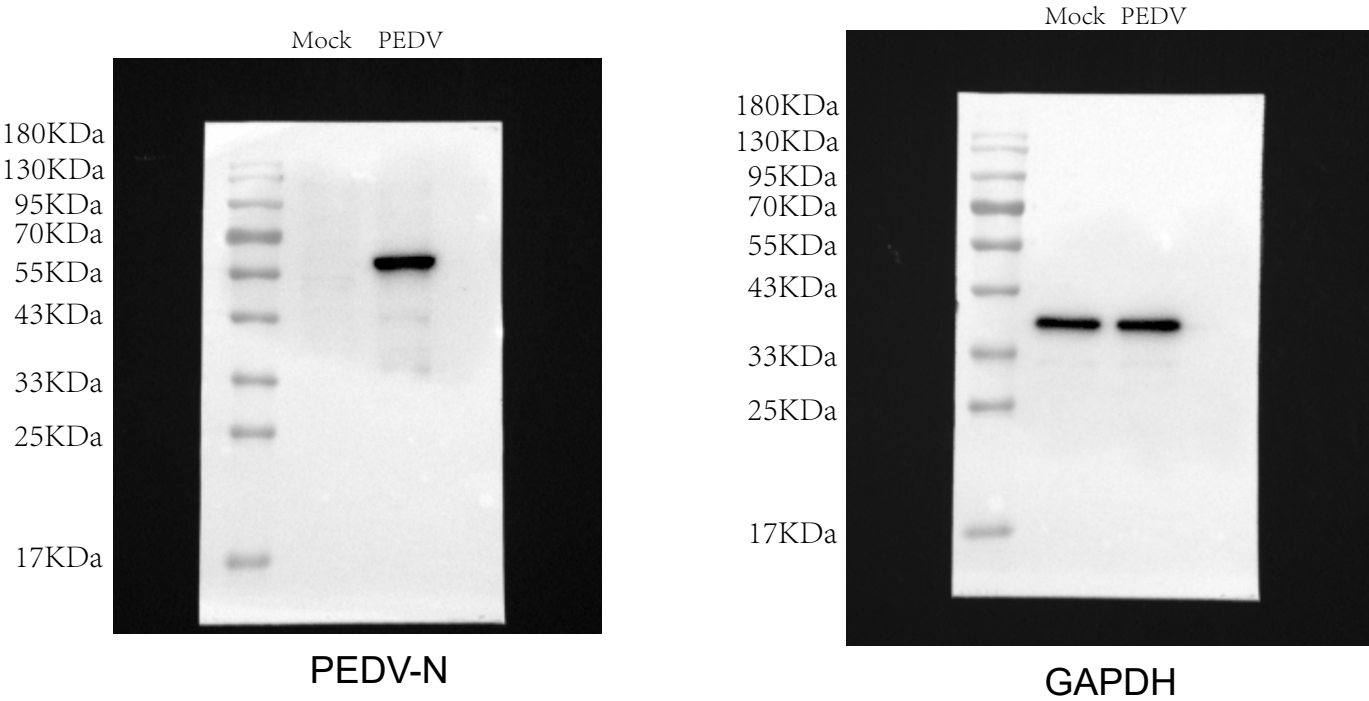

Fig 3B

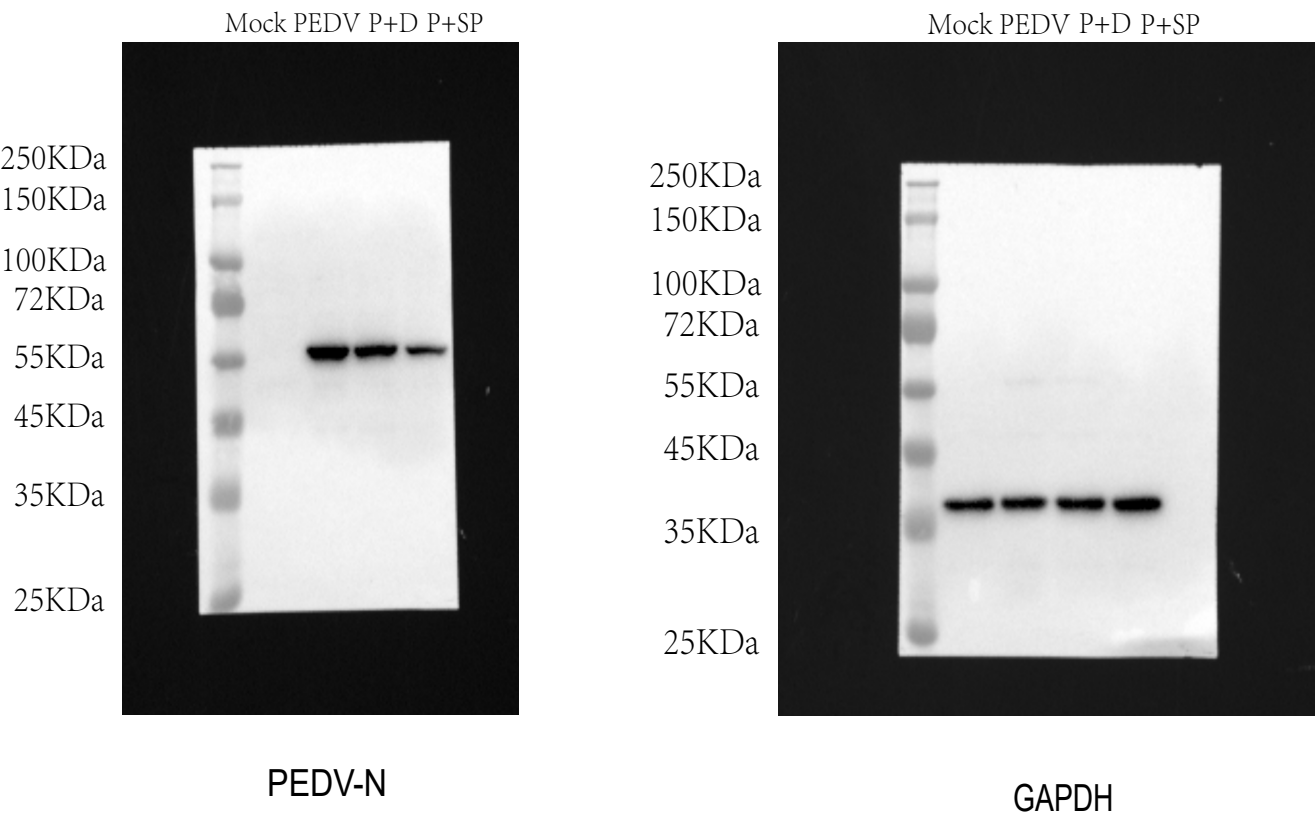

Supplement: Supplementary file 1 — Supplementary Material 1. [file 12917_2024_4052_MOESM1_ESM.pdf]

Supplementary Figure 1 B

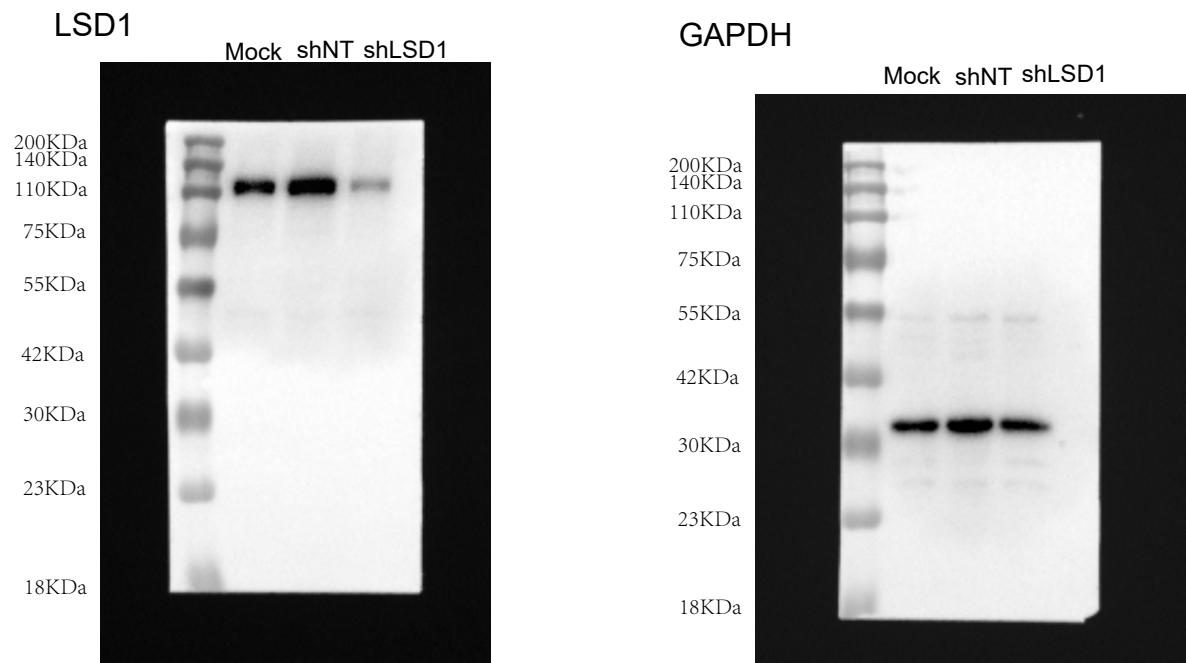

Supplementary Figure 1 C

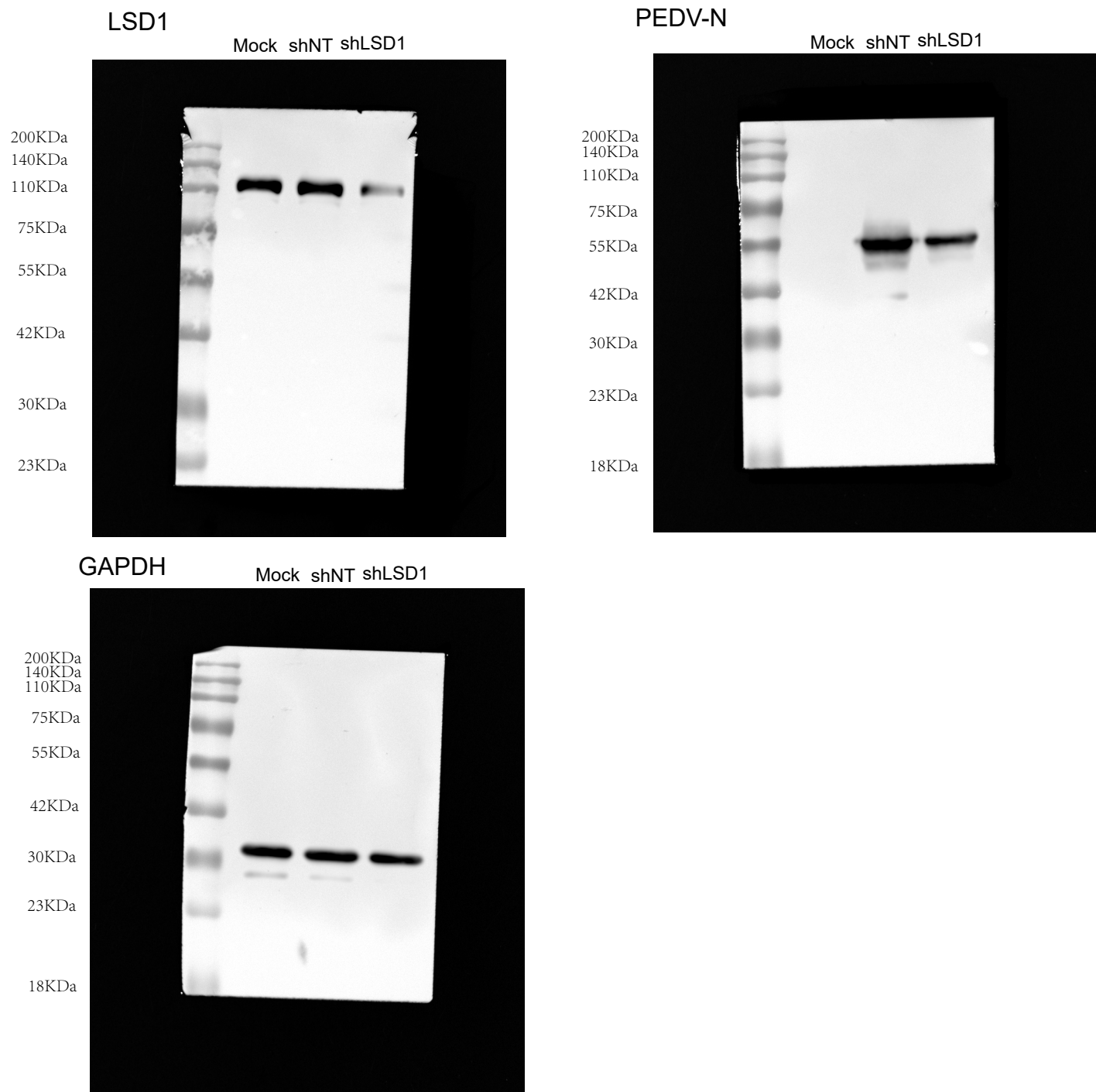

Supplement: Supplementary file 2 — Supplementary Material 2. [file 12917_2024_4052_MOESM2_ESM.pdf]
