# Supplementary material for: SP2509, a specific antagonist of LSD1, exhibits antiviral properties against Porcine epidemic diarrhea virus
Source: BMC Vet Res. 2024 May 10;20:187. doi: 10.1186/s12917-024-04052-5 (PMC11084069; doi:10.1186/s12917-024-04052-5)
Supplement: Supplementary file 3 — Supplementary Material 3. [file 12917_2024_4052_MOESM3_ESM.pdf]

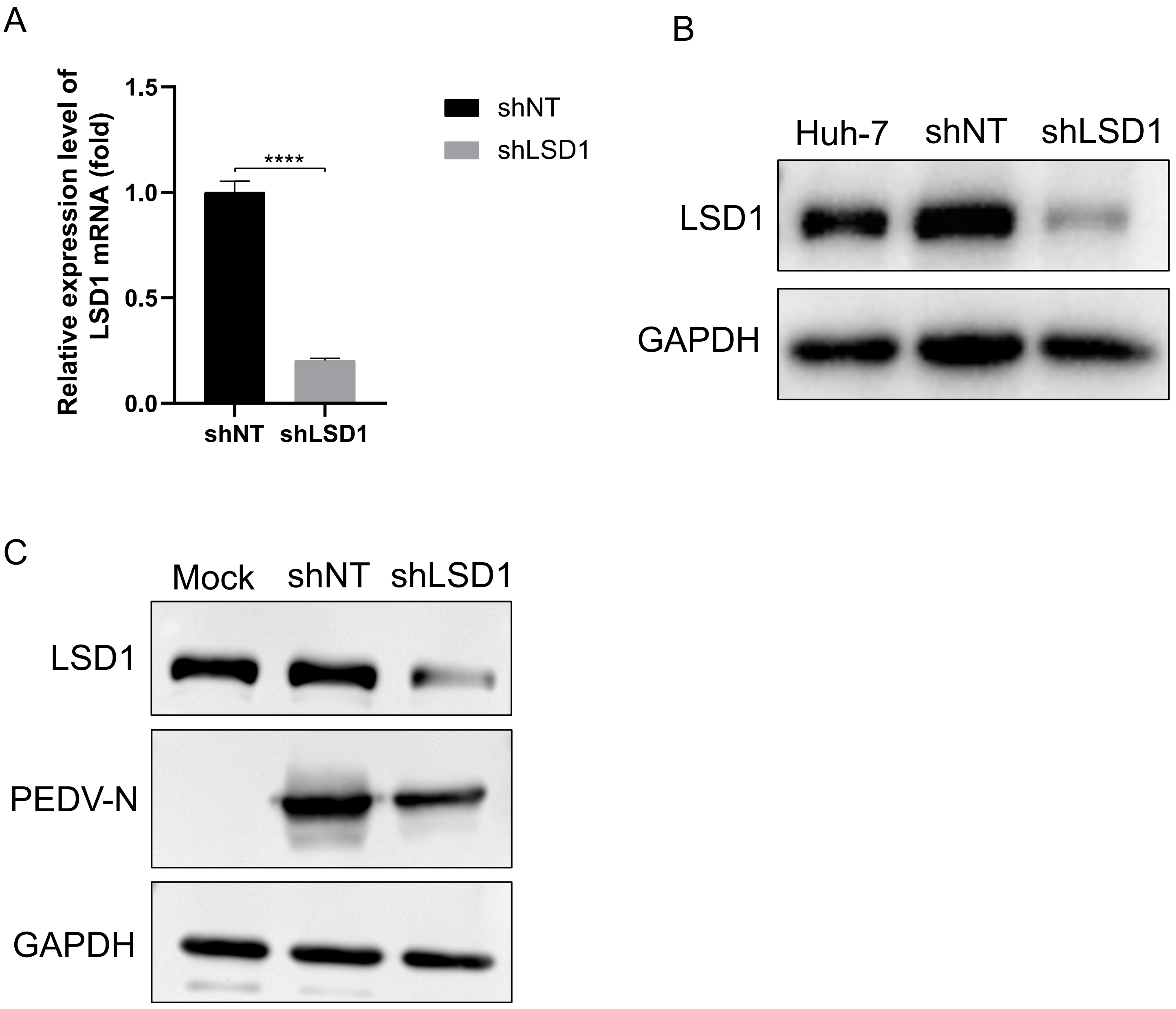

Supplementary Figure 1. Knockdown LSD1 can suppress PEDV replication in Huh-7 cells.
(A) RT-qPCR detected the knockdown efficiency of shLSD1.
(B) The knockdown efficiency of shLSD1 was detected by western blotting.
(C) Western blotting was used to detect the influence of knocking down LSD1 on PEDV.
