# Supplementary material for: SP2509, a specific antagonist of LSD1, exhibits antiviral properties against Porcine epidemic diarrhea virus
Source: BMC Vet Res. 2024 May 10;20:187. doi: 10.1186/s12917-024-04052-5 (PMC11084069; doi:10.1186/s12917-024-04052-5)
Supplement: Supplementary file 4 — Supplementary Material 4. [file 12917_2024_4052_MOESM4_ESM.pdf]

Table S1. The primers for RT-qPCR

Gene	Forward Primer	Reverse Primer
GAPDH	TCACTGCCACCCAGAAGACT	ATGACCTTGCCCACAGCCTT
PEDV-ORF3	GCA CTT ATT GGC AGG CTT TGT	CCATTGAGA AAA GAA AGT GTC GTAG

Table S2. Oligonucleotide sequences shRNA lentiviral vector cloning

shLSD1-F	CCGGCACAAGGAAAGCTAGAAGAAACTCGA GTTTCTTCTAGCTTTCCTTGTGTTTTTT
shLSD1-R	AATTAAAAACACAAGGAAAGCTAGAAGAAA CTCGAGTTTCTTCTAGCTTTCCTTGTG
shNT-F	CCGGCGTGATCTTCACCGACAAGATCTCGAGA TCTTGTCGGTGAAGATCACGTTTTT
shNT-R	AATTAAAAACGTGATCTTCACCGACAAGATCT CGAGATCTTGTCGGTGAAGATCACG
